# Inhibition of CDK8 rescues impaired ischemic fracture healing

**DOI:** 10.21203/rs.3.rs-6458483/v1

**Published:** 2025-05-16

**Authors:** Christina A. Capobianco, Michelle J. Song, Easton C. Farrell, Alexander J. Knights, Karen Kessell, Alexis Donneys, Jeanna T. Schmanski, Luke R. Schroeder, Mengqian Chen, Igor B. Roninson, Yadav Wagley, Tristan Maerz, Kurt D. Hankenson

**Affiliations:** University of Michigan–Ann Arbor; University of Michigan–Ann Arbor; University of Michigan–Ann Arbor; University of Michigan–Ann Arbor; University of Michigan–Ann Arbor; University of Michigan–Ann Arbor; University of Michigan–Ann Arbor; University of Michigan–Ann Arbor; University of South Carolina; University of Michigan–Ann Arbor; University of Michigan–Ann Arbor; University of Michigan–Ann Arbor; University of Michigan–Ann Arbor

## Abstract

Annually, 10% of 15 million bone fractures in the US fail to heal, and fractures with compromised blood flow, i.e. ischemia, are five times more likely to become nonunions. While ischemia is known to impair healing, the cellular and molecular mechanisms underlying this deficiency are unclear. Wild-type mice with surgically-induced ischemia underwent tibia fractures, and single-cell RNA-sequencing was performed on calluses at days 4 and 7 post-fracture. We observed delayed chondrogenic differentiation and upregulation of Cyclin-Dependent Kinase 8 (*Cdk8*) by stromal progenitors and fibroblasts in the ischemic callus. Hypoxia induced CDK8 gene expression in human mesenchymal stromal cells (hMSC), and pharmacological CDK8 inhibition promoted hMSC chondrogenic and osteogenic potential. *In vivo* oral delivery of a CDK8 inhibitor enhanced callus chondrogenesis and mineralization, and improved ischemic fracture healing. Our results suggest that CDK8 impedes stromal cell differentiation and that CDK8 inhibition is a clinically-translatable approach to enhance ischemic fracture healing.

## INTRODUCTION

While fracture repair typically results in complete restoration of functional bone, approximately 10% of fractures in the United States result in a nonunion or malunion, requiring further surgical intervention and treatment ^1–3^. Many factors, including vascular disease, infection, excessive soft tissue damage, advanced age, obesity, nutrition, smoking status, and various endocrine conditions increase the likelihood of fracture nonunion^4^. Specifically, trauma, aging, associated inflammatory dysregulation, and/or smoking place patients at increased risk of ischemia (reduced vascular perfusion) upon fracture^5^. The likelihood of fracture nonunion increases to 46% under ischemic conditions. Despite the clinical recognition of these risk factors, how altered vascularization negatively impact bone regeneration remains largely unexplored.

Ischemic fracture healing is characterized by having reduced chondrocyte and osteoblast differentiation, resulting in smaller and weaker calluses^6^. The chondrocytes and osteoblasts responsible for forming the eventual bony callus via endochondral and intramembranous ossification, respectively, are derived from mesenchymal stromal/progenitor cells (MSC) of the periosteum and endosteum. Extensive research has focused on the molecular regulators of chondrogenesis and osteogenesis of these progenitors^7,8^. While it is well established that ischemia results in decreased callus size, inferior mineralization, and exacerbated fibrosis^6^, the exact nature of this phenotype, especially with regards to the mechanisms driving pathological fibrosis as opposed to chondrogenesis and osteogenesis, which are necessary for bone repair, have yet to be described. Thus, there remains a critical knowledge gap with regards to the cellular and molecular mechanisms that underpin impaired fracture healing due to ischemia.

The objective of this study was to identify, describe, and therapeutically target the cellular and molecular perturbations in the early fracture callus during ischemic fracture healing. We utilized a well-characterized mouse model that mimics vascular injury to assess the effect of ischemia on fracture healing. The model surgically disrupts the femoral blood supply to the lower limb at the time of long bone fracture. We used split barcoding single-cell RNA sequencing (scRNAseq) to analyze fractures at 4- and 7- days post-fracture (dpf), representing the early phase of stromal progenitor recruitment and differentiation. Consistent with previous histological observations of more fibrotic and smaller calluses under ischemic conditions^6^, we observed increased fibroblast presence and delayed chondrogenic cell differentiation in ischemic calluses. Associated with these manifestations, we identified the transcription-regulating kinase *Cdk8* as highly upregulated by stromal cells under ischemic fracture conditions. While this kinase has been shown to negatively regulate cellular differentiation^9,10^, its role in chondrogenesis in the context of fracture healing has not been described. Employing 2D monolayer and 3D organoid cultures, we demonstrated that pharmacological CDK8 inhibition promoted both chondrogenesis and osteoblastogenesis *in vitro*, consistent with CDK8’s role as a negative regulator of cell differentiation. Finally, in two *in vivo* experiments with different treatment timing, we showed that an orally-available CDK8 inhibitor improved fracture healing by enhancing callus chondrogenesis and osteogenesis, establishing a translational therapeutic approach to treating ischemic fractures.

## RESULTS

### Ischemia results in altered stromal cell composition and cell-cell signaling in the early fracture callus

**Figure 1:**
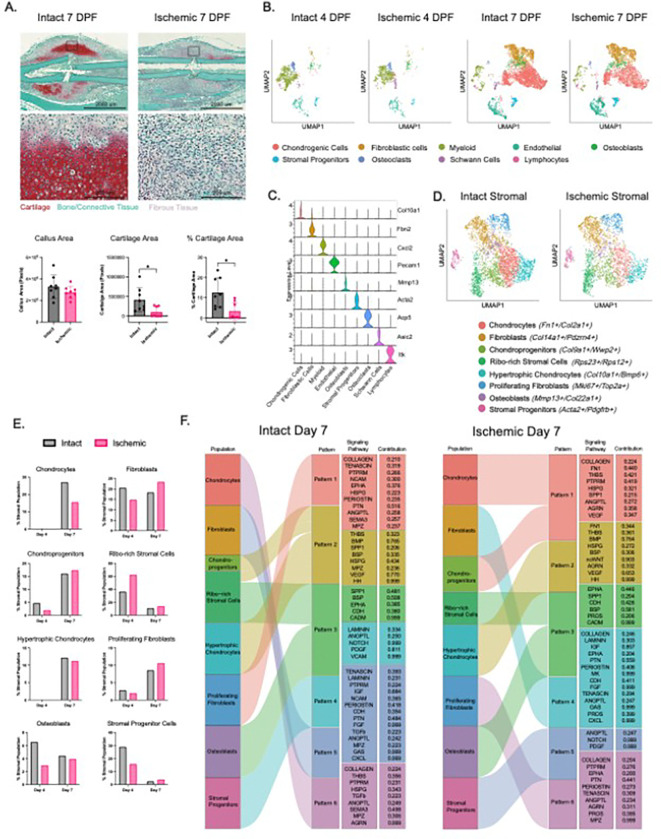
Ischemia results in altered stromal cell composition and cell-cell signaling in the early fracture callus **(A)** Representative Safranin-O histology images of Intact and Ischemic fractured tibias at 7 days post fracture (dpf) (cartilage is red, bone /connective tissue is blue/green, fibrous tissue is purple/white). The fractured bone is outlined in a dashed line. Top panels represent the entire callus (4X stitch, scale bar = 2000 mm), bottom panels represent zoomed in regions depicting cartilage and fibrous tissue (20X stitch, scale bar = 200 mm). Histologic analysis of callus morphology at 7 dpf; % cartilage area, cartilage area, callus area, (mean ± SD, n=8 mice/condition). Normality was assessed via Shapiro-Wilkes. *P<0.05, two-sided t test for Callus Area, Mann-Whitney for non-parametric data for Cartilage Area and % Cartilage Area. **(B)** UMAP plot and cellular annotation from scRNAseq of cells from all fracture conditions. Left to right: Intact 4 dpf, Ischemic 4 dpf, Intact 7 dpf, and Ischemic 7 dpf. **(C)** Violin plots of marker gene expression across the major cell clusters from (B). **(D)** UMAP plot of stromal cell populations combined across timepoints in Intact (right) and Ischemic (left) conditions. Stromal cells were isolated from the data in Fig 1B (Chondrogenic cells, Fibroblastic cells, Osteoblasts, Stromal Progenitors), reclustered, and annotated. (**E**) Proportions of major stromal cell populations in Intact (black) and Ischemic (pink) calluses according to transcriptome-based identification via scRNAseq at 4 dpf and 7 dpf. (**F**) River plot from CellChat crosstalk analysis demonstrating outgoing communication pathways by major stromal cell clusters from (D) at 7 dpf in Intact and Ischemic conditions. The gene pathways comprising each pattern and contribution score are listed to the right for each.

Our group and others have demonstrated that ischemic fracture conditions lead to impaired callus formation and mineralization^6^. Specifically, ischemic calluses exhibit reduced bone volume and increased fibrous tissue, suggesting dysregulation of endochondral ossification^6^. To characterize the cellular and molecular effects associated with this phenotype, we induced distal hindlimb ischemia in mice by surgical ligation and resection of the left femoral artery (“Ischemic”), or kept vasculature intact (“Intact”), followed by tibial fracture induction in all mice using a three-point bending apparatus. At 7 days post fracture (dpf), representing the early phase of stromal expansion after fracture, we observed a pronounced decrease in cartilage in the ischemic calluses compared to intact callus (Fig. 1A). However, consistent with Lu et al. ^6^, there was no difference in callus size, emphasizing the amount of fibrotic tissue that comprises the ischemic fracture callus. scRNAseq of fracture callus under Intact and Ischemic conditions revealed a time-dependent emergence of stromal, immune, neuronal-related (schwann cells), and endothelial cells in the fracture calluses. (Fig. 1B–C, **Fig S1–2**). A profound transition occurred between 4 to 7 dpf, during which the dominance of immune cells at 4 dpf (myeloid, osteoclasts, and lymphocytes) gave way to a pronounced stromal expansion at 7 dpf (chondrogenic, fibroblastic, stromal progenitors, and osteoblasts) (Fig. 1B). We also observed an increase in the endothelial cells by 7 dpf (Fig. 1B). Based on their transcriptional profiles, stromal cells were broadly grouped into chondrocytes, osteoblasts, fibroblastic, and a progenitor subset (**Fig. S2A**). Isolated analysis of only stromal cells, defined as *Prrx1*^*+*^*/Runx2*^*+*^*/Cdh11*^*+*^*/Postn*^*+*^*/Fn1*^*hi*^*/Col1a2*^*hi*11^, revealed eight functionally unique subsets. These encompassed discrete chondrogenic, osteogenic, and fibroblastic populations that varied across timepoints and injury conditions (Fig. 1D, **Fig. S3A-C**). At 4 dpf, the stromal progenitor population (*Acta2*^*+*^*/Pdgfrβ*^*+*^) ^12^, fibroblasts (*Co/14a1*^*+*^*/Pdzrn4*^+^) ^13,14^, proliferating fibroblasts (*Mki67*^*+*^*/Top2a*^+^) ^15^, chondroprogenitors (*Co/9a1*^*+*^*/Wwp2*^+^) ^16,17^, ribosome-enriched stromal cells (*Rps23*^*+*^*/Rps12*^+^), and osteoblasts (*Mmp13*^*+*^*/Col22a1*^+^) ^18,19^ were identified (Fig. 1E, **Fig S3B**), followed by the further emergence of chondrocytes (*Fn1*^*+*^*/Col2a1*^+^) ^20,21^ and hypertrophic chondrocytes (*Col10a1*^*+*^*/Bmp6*^+^) ^22,23^ at 7 dpf (Fig. 1E, **Fig. S3B**). Consistent with histological results, ischemic callus had 57.6% fewer chondroprogenitors at 4 dpf and 42.4% fewer chondrocytes, 7.5% fewer hypertrophic chondrocytes, 11.1% fewer osteoblasts, and 27.5% more fibroblasts at 7 dpf, further evidencing a more fibrotic and less chondrogenic callus under ischemic conditions (Fig. 1D–E). Ischemic fractures additionally had 45.3% fewer stromal progenitors at 4 dpf suggestive of potentially decreased stromal recruitment and proliferation at the fracture site (Fig. 1E).

We next sought to assess how ischemia alters the signaling patterns of stromal cells using CellChat analysis to quantitatively infer cell to cell communication^24^. The outgoing (Fig. 1F, S4) and incoming (**Fig. S5**) communication pathways at 7 dpf depicted similar patterns between Intact and Ischemic conditions with disturbances in key signaling pathways indicative of increased fibrosis under Ischemic conditions. Specifically, pathways involved in bone metabolism and matrix development including Semaphorin 3 (Sema3) and transforming growth factor beta (Tgfβ) outgoing signaling pathways were exclusive to the Intact chondrocytes and proliferating fibroblasts. However, under Ischemic conditions, fibronectin 1 signaling (Fn1) was upregulated by the chondrogenic populations, emphasizing altered matrix deposition (Fig. 1F). Additionally, in Intact calluses, stromal progenitor cells exhibited significant vascular cell adhesion molecule (Vcam), and Laminin outgoing signaling pathways in addition to Notch and platelet derived growth factor (Pdgf), however Laminin and Vcam were not predicted as significant pathways under Ischemic conditions for this population (Fig. 1F). Incoming signaling pathways were similarly described for key chondrogenic regulators including Tgfβ, heparan sulfate proteoglycans (Hspg), and Thbs signaling to chondrogenic populations, which were not observed under Ischemic conditions (**Fig S5**). These findings demonstrate at the single-cell level that ischemia results in a more fibroblast-rich callus, impaired stromal function, and a signaling program of inferior chondrogenesis and ECM production.

### Ischemia promotes increased T cell recruitment during the inflammatory phase of fracture healing

Consistent with prior literature, we established that the early fracture callus (4 dpf) is largely comprised of macrophages and neutrophils ^25^. By 7 dpf, the percent of the immune population within the callus shifts towards an increase in dendritic cells and T cells (**Fig S6–7**). scRNAseq analysis of immune populations was corroborated by immunophenotyping using flow cytometry to distinguish macrophages (CD4+/F480+/CD11b+), neutrophils (CD45+/F480−/Ly6G+), dendritic cells (CD45+/F480−CD11c+), and T cells (CD45+/CD3+) (**Fig S6C-D**). Interestingly, we identified a ribosome-rich immune population that differentially expressed the hematopoietic stem cell marker *Cd34*, as well as the GO term “positive regulation of hematopoietic progenitor cell differentiation”, these cells are most prevalent at 4 dpf and diminished by 7 dpf, and could represent hematopoietic stem cells (**Fig S7**). The greatest observed difference in immune cell populations between Intact and Ischemic calluses was a two-fold increase in T cell content as shown by flow cytometry (**Fig S6D**). In addition, gene ontology analysis demonstrated decreased acute inflammatory response, leukocyte aggregation/degranulation, and T cell cytokine production indicative of immune cell dysregulation at 4 dpf, concordant with decreased terms associated with extracellular matrix organization by 7 dpf (**Fig S8**). These results demonstrate that alongside alterations to stromal cell content, ischemia also perturbs immune cell composition of early fracture callus, marked by an increase in T cells and dysregulated T cell function.

### Ischemic calluses exhibit delayed and more fibroblastic stromal progenitor differentiation

**Figure 2:**
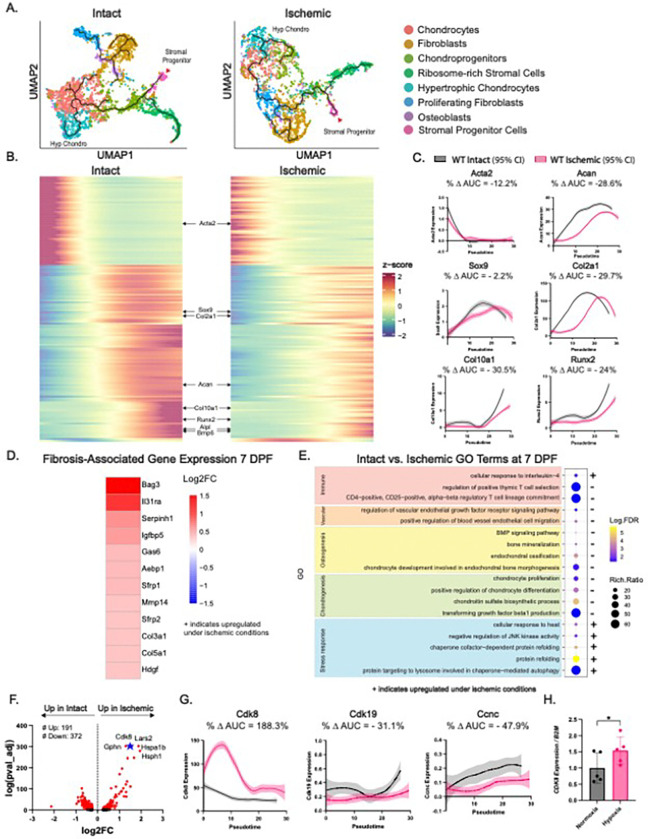
Ischemic calluses exhibit delayed, fibroblastic stromal progenitor differentiation with associated cellular stress and *Cdk8* upregulation **(A)** Cellular trajectory analysis via Monocle3 - Intact (left) and Ischemic (right) conditions. Cells are color-coded according to their stromal cluster from Fig 1D. Red arrowhead denotes the αSMA+ progenitors. **(B)** Heatmap of relative expression (depicted by z-score) of the top 138 genes with the greatest pseudotime-dependent expression changes in Intact (left). X-axis is increasing pseudotime, originating at αSMA+ progenitors, ending at hypertrophic chondrocytes. Hierarchical clustering was performed to bin genes in the y-axis. The same genes, same order, were analyzed in Ischemic along pseudotime (right). **(C)** Pseudotime regression plots of normalized expression of progenitor- and chondrogenesis-relevant genes in Intact (black) and Ischemic (pink) as a function of increasing pseudotime, along the respective trajectories in (A). Shaded areas represent 95% confidence intervals. % change in the area under the curve (AUC) of expression between Intact and Ischemic is shown on each plot. **(D)** Heatmap of relative gene expression of selected fibroblastic genes upregulated in Ischemic calluses at 7 dpf **(E)** Bubble plot of pathway enrichment analysis (GO:Biological Processes via PantherDB) of differentially-expressed genes in all stromal cells between Intact and Ischemic. Positive sign (+) indicates upregulation in Ischemic. Terms are color-coded according to theme. **(F)** Volcano plot of differentially expressed genes (Padj < 0.05 in red) between Intact and Ischemic at 7 dpf. Positive fold changes indicate upregulation in Ischemic. Top 5 most differentially expressed genes according to Padj are labeled; blue star indicates *Cdk8*. Number of differentially upregulated and downregulated genes according to Padj < 0.05 are listed. **(G)** Pseudotime regression plot of normalized *Cdk8, Cdk19,* and *Ccnc* expression by stromal cells in Intact (black) and Ischemic (pink) as a function of increasing pseudotime, along the respective trajectories in (A) with 95% confidence intervals and % change in AUC. **(H)** *CDK8*gene expression by primary hMSCs cultured in normoxia (20% oxygen) or hypoxia (1% oxygen) for 72 hours via quantitative RT-PCR (qPCR). N = 5 unique donors. Data represented as mean ± SD. Normality was assessed via Shapiro-Wilkes. *P<0.05, two-tailed paired t-test.

The coordinated differentiation of stromal progenitors into chondrogenic and osteoblastic cell populations is an essential prerequisite for fracture healing. Specifically, αSMA+ (*Acta2+*) progenitors expand following fracture and transition into osteoblasts and chondrocytes^12^. Osteoblasts produce bone directly via intramembranous bone formation, and chondrocytes form a cartilage intermediate that ultimately undergoes endochondral ossification to form bone. To assess whether ischemia negatively altered stromal progenitor differentiation as part of the inferior callus phenotype, we performed scRNAseq-based cellular trajectory analysis using Monocle 3^26^. This was conducted in Intact and Ischemic conditions independently, withαSMA + progenitor cells as the trajectory origin. In both Intact and Ischemic conditions, we observed a trajectory originating in αSMA + cells, transitioning through multiple stromal lineages, and terminating in hypertrophic chondrocytes (*Col10a1+/Bmp6+*) (Fig. 2A). We also identified a branching trajectory from αSMA + cells to ribosome gene-enriched stromal cells. These cells are indicative of metabolically active protein synthesis and a stressed cell state^27^. In the Intact condition, this trajectory encompassed a direct transition of the αSMA + progenitor state to chondroprogenitors, which terminated in hypertrophic chondrocytes via a chondrocyte intermediate (Fig. 2A). However, under ischemia, this trajectory involved a fibroblast-like (*Col14a1+/Pdzrn4+*) intermediate state between the αSMA + origin and chondrogenic trajectory endpoint (Fig. 2A). To characterize the molecular alterations of the Ischemic trajectory, we extracted all genes with pseudotime-variant expression in the Intact condition, performed unsupervised hierarchical clustering, and compared their profiles to the Ischemic condition. This revealed massive perturbation to the transcriptional dynamics of chondrogenic differentiation (Fig. 2B). Based on area-under-the-curve (AUC) analysis of genes involved in chondrogenesis along pseudotime, *Lmod1, Tgm2, TagIn, Myh11, and Adamts4* exhibited the greatest differences in pseudotime-dependent gene expression between Intact and Ischemic conditions (**Table S1**). *Sox9* and *Runx2*, as well as genes encoding cartilage matrix constituents (*Col2a1, Col10a1,* and *Acan*) demonstrated a clearly delayed and diminished pseudotime-dependent expression pattern in Ischemia compared to Intact (Fig. 2C). Targeted analysis of DEGs upregulated in Ischemic calluses at 7 dpf revealed overexpression of fibrosis-relevant genes *including Igfbp5*^28^, *Sfrp1*^29^, *Sfrp2*^30^, *Aebp1*^31^, *Bag3*^32,33^, *II31 ra*^34,35^, *Col3a1*^36^, *Col5a1*^37^, *Mmp14*^38^, *Hdgf*^39^, *and Serpinh1* ^40,41^ (Fig. 2D). These results support that the more fibroblastic callus observed in ischemic conditions is associated with the failure of stromal progenitors to effectively transition to chondrocytes.

### Cdk8 upregulation is associated with impaired stromal differentiation and cellular stress in Ischemic fractures

Since stromal differentiation trajectories in Ischemic conditions were markedly perturbed, we performed unbiased differential gene expression analysis and determined enriched biological pathways of all stromal cells between Intact and Ischemic conditions. There were 563 DEGs at P_adj_ < 0.05 (191 up, 372 down) between Intact and Ischemic stromal cells, which were associated with biological pathways related to immune response, vascularization, osteogenesis, chondrogenesis, and stress response. Within the ischemic population we observed diminished T cell regulation, endothelial cell migration, endochondral ossification, and chondrocyte proliferation and differentiation (Fig. 2E). Conversely, functions associated with stress response - protein refolding and cellular response to heat were enriched under ischemia. In Ischemic calluses, we also observed increased response to IL-4, which can play a role in fibrosis^42,43^ (Fig. 2E).

Among the top upregulated genes in stromal cells under Ischemic conditions was the gene *Cdk8*, encoding cyclin dependent kinase 8 (CDK8). CDK8 and its paralog CDK19 are enzymatic components of the regulatory CDK module, which associates with the transcriptional Mediator complex, and CDK8 regulates transcription positively or negatively in a context-specific manner^44,45^. CDK8 has been demonstrated to impede cell reprogramming and differentiation across multiple cell types, including fibroblast differentiation to hepatocytes and adipocytes^46^. In all stromal cells combined at 7dpf, DEG analysis demonstrated a 2.5-fold increase (Padj =5.90e-303) in *Cdk8* expression in Ischemia compared to Intact conditions (Fig. 2F). We also observed the upregulation of multiple heat shock proteins (*Hspa1b, Hspa1a, Hsph1, Hsp90aa1*), interpretable as indicators of cellular stress^47^. CDK8 has also been shown to be required for heat shock protein activation during states of cell stress^48,49^. Analysis of *Cdk8* expression along the stromal progenitor trajectory demonstrated markedly greater *Cdk8* expression in cells from Ischemic calluses, notably at low pseudotime values (i.e. early progenitor states) (Fig. 2G). AUC analysis further demonstrated markedly higher cumulative *Cdk8* expression (% Δ AUC = 188.3%) by stromal cells under Ischemic conditions (Fig. 2G). In contrast, *Cdk19*, a paralog of *Cdk8* that can substitute for *Cdk8* in transcriptional regulation^50^, was not overexpressed in Ischemia. *Cdk19* and *Ccnc*, an activator of CDK8 activity, were lowley expressed in both Intact and Ischemic conditions (Fig. 2G). Corroborating the potential role of ischemia in modulating *Cdk8* expression in stromal progenitors, primary human mesenchymal stromal cells (hMSC) cultured under hypoxic conditions exhibited upregulation of *CDK8* expression (Fig. 2H). These findings demonstrate that ischemia results in markedly impaired stromal progenitor differentiation, associated with a signature of cellular stress and the upregulation of CDK8, which may act as a molecular brake of differentiation^46^.

### Inhibition of CDK8 improves chondrogenic and osteogenic differentiation

**Figure 3:**
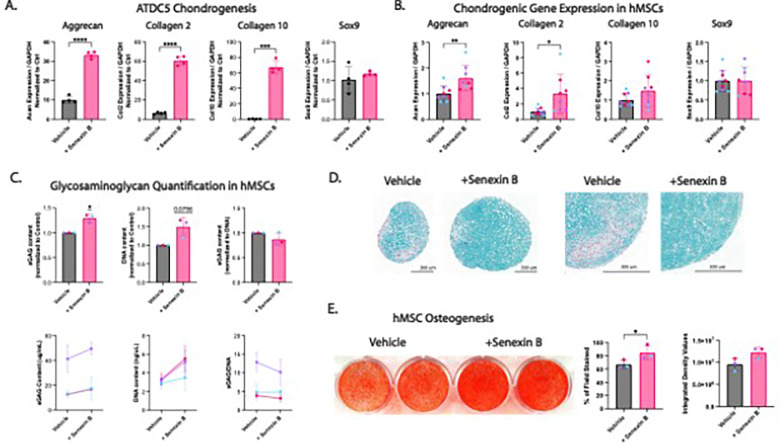
Inhibition of CDK8 improves chondrogenic and osteogenic differentiation **(A)** Gene expression of chondrogenesis-relevant genes, via qPCR, by ATDC5 cells treated with either Vehicle (black) or 400 nM Senexin B (pink) during 14 days of chondrogenic differentiation. Individual data points represent n=4 technical replicates. **(B-D)** Gene expression of chondrogenesis-relevant genes via qPCR (B), glycosaminoglycan content via DMMB assay (C), and Alcian blue-stained histologic sections of primary hMSC-based chondrogenic pellets treated with Vehicle or Senexin B during 14 days of chondrogenic differentiation. **(B)** Gene expression of chondrogenesis-relevant genes was analyzed from the RNA of 3 pooled pellets for each donor (n=3 unique donors, n=2–3 technical replicates per donor). **(C)** Two pooled chondrogenic hMSC pellets per unique donor were digested and glycosaminoglycan and DNA content were quantified. sGAG content is expressed as both unnormalized (bottom row) and normalized to the Vehicle control. sGAG content was further normalized to DNA content (n=3 unique donors). In normalized plots, a one sample t test is applied. **(D)** Chondrogenic hMSC pellets were paraffin processed, sectioned (5 μm), and stained with 1% Alcian Blue in 3% Acetic Acid and Nuclear Fast Red stain. Sections were imaged at 10X using brightfield microscopy. Scalebar = 300 um. (n = 3 unique donors, n = 2–3 technical replicates) Each color represents a unique biologic donor. **(E)** Osteogenesis was induced in 2D cultured hMSCs treated with Vehicle and 400 nM Senexin B and analyzed for mineralization with Alizarin red S staining after 10 days (n = 3 unique donors). Data represented as mean ±SD. Normality was assessed via Shapiro-Wilkes. *P<0.05, **P<0.01, ***P<0.001, ****P<0.0001, two-sided t test, Welch-corrected 2 sided-t test for plots with nonequivalent variance in 3A, one-sided t test for normalized plots in 3C, Wilcoxon test for non-parametric data in 3C sGAG Content (ug/mL) an 3E Integrated Density Values.

To probe the potential role of CDK8 in mediating stromal progenitor differentiation, we next employed the CDK8-specific inhibitor Senexin B^51–53^ in the context of *in vitro* chondrogenesis of murine ATDC5 cells and hMSC. Treatment of ATDC5 cells with Senexin B resulted in dramatic increases in the expression of *Acan, Col2a1,* and *Col10a1* during monolayer differentiation, with minimal effects on *Sox9* expression (Fig. 3A). Three-dimensional hMSC chondrogenesis in the presence of Senexin B for 14 days also resulted in greater expression of *ACAN* and *COL2A1*, and Senexin B-treated pellets exhibited ~ 25% greater sulfated glycosaminoglycan (sGAG) content (Fig. 3B–C). Histomorphometrically, Senexin B-treated pellets were larger in size and exhibited more robust and homogeneous sGAG content, with fewer sGAG-devoid regions compared to the control (Fig. 3D). Consistent with the larger pellet size, the DNA content was also increased by Senexin B treatment, suggesting that inhibition of CDK8 may promote hMSC proliferation (Fig. 3C). Although no difference in total sGAG content was observed between Vehicle and Senexin B-treated samples after normalization to total DNA content (Fig. 3C), histomorphometric analysis suggested superior chondrogenesis when CDK8 kinase activity is inhibited. Lastly, we treated hMSCs with Senexin B and confirmed that CDK8 inhibition enhanced *in vitro* mineralization in the presence of BMP2 (Fig. 3E), corroborating prior findings^54^. Taken together, these results indicate that CDK8 activity impedes the chondrogenic and osteogenic differentiation capacities of stromal progenitors, and its pharmacological inhibition with Senexin B enhances chondrogenesis and osteoblastogenesis.

### Oral pharmacologic CDK8 inhibition improves ischemic bone healing in vivo

**Figure 4:**
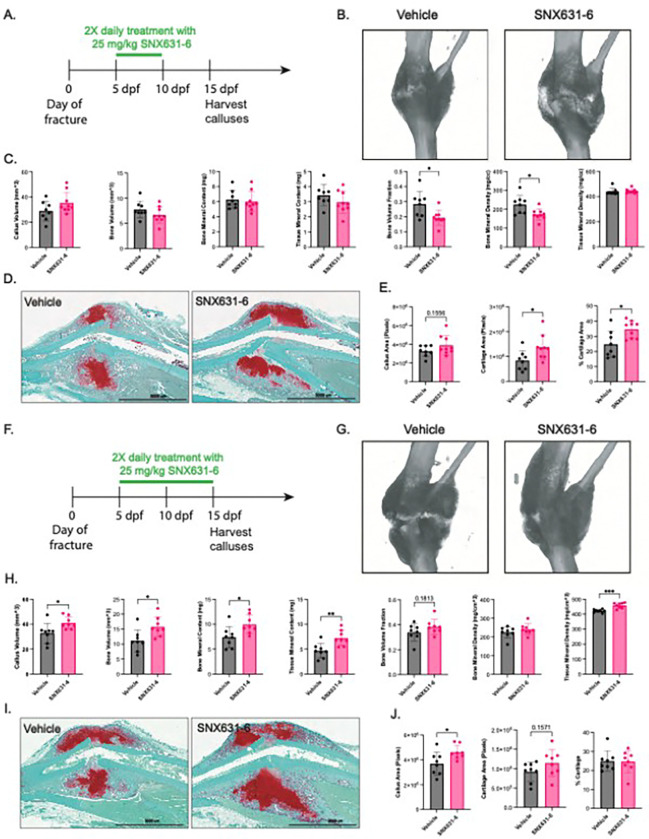
Oral treatment with SNX631-6 leads to increased callus size and bone content by 15 days post fracture in ischemic tibia fractures. **(A)** Timeline of oral gavage treatment from 5–10dpf with 25mg/kg SNX631-6. **(B)** Representative mCT images of fractured tibiae 15 dpf from vehicle (30% Propylene Glycol, 70% PEG-400 twice daily) and SNX631-6 (25 mg/kg twice daily) groups treated from 5–10 dpf. **(C)** mCT analysis of callus morphology at 15 dpf from groups treated 5–10 dpf; bone volume, bone mineral density, tissue mineral density, callus volume, bone volume fraction, bone mineral content, tissue mineral content (n = 8 vehicle, n = 9 SNX631-6). **(D)** Representative histology images of vehicle and SNX631-6 fractured tibias at 15 dpf from groups treated 5–10dpf, (4X stitch, scalebar = 3000 mm). **(E)** Histologic analysis of callus morphology at 15 dpf from groups treated 5–10 dpf; % cartilage area, cartilage area, callus area, (n = 8 vehicle, n = 9 treatment, average of 3 technical replicates/callus). **(F)** Timeline of oral gavage treatment from 5–15 dpf with 25 mg/kg SNX631-6. **(G)** Representative mCT analysis of callus morphology at 15 dpf from groups treated 5–15dpf. **(H)** mCT analysis of callus morphology at 15 dpf from groups treated 5–10 dpf. (I) Representative histology images of vehicle and SNX631-6 fractured tibias at 15 dpf from groups treated 5–15dpf. (J) Histologic analysis of callus morphology at 15 dpf from groups treated 5–15dpf, (4 X stitch, scalebar = 3000 mm). Data represented as mean ± SD. Normality was assessed via Shapiro-Wilkes. *P<0.05, **P<0.01, two-sided t test.

To confirm the functional importance of CDK8 *in vivo* and to assess whether pharmacological CDK8 inhibition is a viable therapeutic strategy to improve fracture healing, we sought to therapeutically block CDK8 to rescue impaired healing of ischemic fractures. We utilized the CDK8 inhibitor SNX631–6, an orally available pharmaceutical with ~ 10 times greater potency than Senexin B^55^. We first confirmed that SNX631-6 recapitulated the beneficial effects of Senexin B on chondrogenesis in vitro. Chondrogenic ATDC5 cells treated with SNX631-6 exhibited similar upregulation of chondrogenic genes, including *Collagen 2 (Col2a1), Collagen 10 (Col10a1), Aggrecan (Acan)*, but not of Sox9 **(Fig S9)**. Next, we employed two treatment regimens to assess how orally-administered SNX631-6 (25mg/kg twice daily) impacted the early chondrogenic phase (treatment 5–10 dpf) (Fig. 4A–E) and the chondrogenic-to-osteogenic phase (treatment 5–15 dpf, Fig. 4F–J) in the healing of ischemic murine fractures. Treatment during 5–10 dpf demonstrated a slight increase in μCT-based callus volume but not enhanced callus bone formation (Fig. 4B–C). Histological analysis corroborated this trend of increased volume and depicted significantly increased callus cartilage content, by both total and proportional area (Fig. 4D–E). These *in vivo* findings corroborate our observed *in vitro* effects of CDK8 inhibition to improve chondrogenesis (Fig. 3A–D).

CDK8 inhibition via SNX631-6 treatment during 5–15 dpf improved fracture healing by increasing μCT-based bone volume, total callus volume, bone mineral content, tissue mineral content, and tissue mineral density, as compared to control (vehicle) treatment (Fig. 4G–H). Histological analysis corroborated significantly larger callus size in SNX631-6-treated mice, with no difference in the proportion of total area of cartilage (Fig. 4I–J). Increased bone volume with treatment during the chondrogenic-to-osteogenic transitionary period is consistent with the findings in Fig. 3E and demonstrates that when osteogenic cues are present, CDK8 inhibition improves bone formation.

## DISCUSSION

Insufficient blood supply leads to reduced delivery of oxygen and nutrients to cells, a clinically unfavorable condition that has long been recognized as a cause of nonunion or malunion fractures^56^. Ample evidence exists showing that the ischemic callus forms less bone and overall tissue volume, an effect that persists throughout the healing process^5,6^. Despite these observations of impaired callus formation in ischemic fracture healing, the cellular and molecular mechanisms underpinning this phenotype have not been well characterized. We analyzed the cellular milieu underlying impaired ischemic conditions and discovered impaired stromal progenitor differentiation associated with upregulated *Cdk8* expression.

Fibrotic healing, rather than chondrogenic and osteogenic healing, is a well-described consequence of multiple impaired fracture conditions, including ischemia^6,57,58^. Using scRNAseq-based trajectory analysis and unbiased derivation of the transcriptional programs underpinning stromal progenitor differentiation, we observed aberant stromal differentiation towards a fibroblastic intermediate state in ischemic conditions. Whereas control (Intact) calluses transitioned from aSMA/*Acta2* + progenitors to chondroprogenitors and hypertrophic chondrocytes, Ischemic calluses exhibited a clear fibroblastic intermediate cell state characterized by high expression of Matrix metallopeptidase 14, Collagen 3, and Collagen 5 with diminished expression of canonical chondrogenic genes such as Aggrecan, Collagen 2, and Collagen 10. Our study characterized the cellular identity and differentiation trajectory of the cells underpinning fibrotic healing in ischemic fracture calluses, facilitating additional future studies targeting these cells.

We demonstrated that impaired stromal progenitor differentiation during ischemic fracture healing is associated with upregulation of *Cdk8*, but not of its paralog *Cdk19*. Trajectory analysis demonstrated that Ischemic calluses have severely reduced and delayed chondrogenic gene expression that coincides with greater expression of *Cdk8*, notably in early progenitor states. Our findings corroborate the work by other groups that CDK8 inhibits fibroblast reprogramming and differentiation^46^. Using selective pharmacologic inhibitors of CDK8/19, we demonstrated that CDK8 impedes chondrocyte and osteoblast differentiation, resulting in reduced chondrogenesis and endochondral ossification that comprise impaired healing conditions both *in vitro* and *in vivo*. Most importantly, we confirmed that *in vivo* CDK8 inhibition using an orally-available drug improved callus formation, with enhanced chondrogenesis and callus bone formation, consistent with improved healing. These are clinically relevant results as they suggest that oral treatment with CDK8 inhibitors may be able to modulate impaired fracture healing, addressing a major unmet clinical need.

Prior literature has demonstrated that *Cdk8* is upregulated under aging-associated osteoporosis and that genetic inhibition of *Cdk8* rescued the impaired senescent MSC phenotype in aged mice^59^. In accordance with our work, pharmacologic inhibition of CDK8/19 using CDK8/19 inhibitors have both been shown to inhibit osteoclast activity and upregulate osteoblast activity in a mouse model *in vitro* and in a rat model upon local (intra-bone) administration^54^. Here, we demonstrated that *Cdk8* broadly inhibits differentiation of both osteogenesis and chondrogenesis and that impaired bone healing can be rescued by systemic administration of an orally available CDK8/19 inhibitor.

Through our two treatment regimens of CDK8 inhibition during the early chondrogenic callus formation and the chondrogenic to osteogenic transition in the ischemic fracture environment, we see definitive increases in cell differentiation to chondrocytes (day 5–10) and enhanced osteogenesis (day 5–15), respective to the cues present in the environment at these timepoints. This is consistent with our *in vitro* work. While we observed a reduction in bone volume fraction and bone mineral density in the day 5–10 treatment group, there was comparable bone volume overall, suggesting that these decreased proportions are due to increased callus size. We anticipate that if a later time period was studied, that the greater cartilage in this group would undergo mineralization, thus resulting in an overall stronger callus. We additionally observed an increase in bone formation in the longer day 5–15 treatment group, however, it is unclear whether this is due to direct MSC osteogenesis or hypertrophic chondrocytes undergoing endochondral ossification. Either explanation is possible given that our *in vitro* results support both a direct osteogenic effect of CDK8 inhibition and an expansion of cartilage. We saw increases in hypertrophic chondrocyte marker *Col10a1* indicative of expedited chondrocyte differentiation towards hypertrophy. Previous studies have shown that *Cdk8* inhibition through *Cdk8* RNAi maintains SMAD phosphorylation in the BMP and TGFβ signaling pathways long after removal of the stimuli, potentially driving our observed increase in differentiation^60^.

CDK8 is a unique pharmacologic target in impaired fracture healing with clinically relevant, orally-bioavailable, non-toxic drugs already in cancer clinical trials ^61–64^. Our study demonstrates a translational therapeutic strategy for improving impaired fracture healing outcomes through noninvasive means that would complement surgical measures. Additional *in vivo* experimentation focused on elucidating specific transcriptional effects of CDK8 will be required to assist in understanding its role in callus formation and mineralization. Future *in vitro* differentiation and crosstalk studies will expand upon and corroborate mechanistic interrogation of how CDK8 functions to promote differentiation.

## METHODS

### Animal studies

The study was designed under the guidance of a university-wide AAALAC-accredited laboratory animal medicine program. Protocols used in this study were reviewed and approved by the University of Michigan Animal Care and Use Committee (IACUC).

C57 BI/6 mice were purchased from Jackson Labs (Bar Harbor, ME). All mice were allowed at least two weeks’ time to accommodate to the pathogen-free vivarium on a 12-hour light/dark cycle at the University of Michigan prior to studies. All animals were allowed ad libitum access to food and water. Mice were socially housed (3–5 mice per cage) in the same vivarium.

Mice were randomly assigned to control or experimental group. For all surgical and fracture procedures, mice were anesthetized using inhaled isoflurane (4% for induction; 2–4% for maintenance) and given a single dose of Ethiqa XR (extended-release Buprenorphine) subcutaneously (SC) for analgesia (3.25 mg/kg). Animals were humanely euthanized through carbon dioxide inhalation, followed by cervical dislocation for confirmation of death. All procedures were approved by the Institutional Animal Care and Use Committee (IACUC).

Tibial fractures were performed at 18(±2) weeks of age. A three-point bend apparatus was utilized to generate bilateral tibia fractures, as previously described . To do so, 30-gauge needles were first inserted into the intramedullary space of both tibiae to stabilize each bone. This was radiographically confirmed prior to clipping the needles flush with the proximal tibia. A 272-gram weight was then dropped onto the tibia inducing a traumatic fracture directly distal to the tibiofibular junction as previously described by our lab^65,66^. This was done consecutively in both tibiae, and a transverse fracture pattern was confirmed through radiography.

Alternatively, mice underwent ischemic unilateral left tibia fractures. Prior to incision, 0.5% Bupivacaine was injected locally. Under anesthesia, a 15 mm incision was created proximal to the knee along the length of the femur in the left limb. The femoral artery was isolated and a 2 mm section was ligated using 8–0 nylon sutures. The artery was then resected, leaving a 2 mm gap, inducing ischemia in the distal limb ^6^. Immediately following arterial resection, left tibia are pinned and fractured as described above.

### CDK8 inhibitor treatment

Male C57 BI/6 mice at 18 weeks old underwent ischemic left tibia fractures as described. Mice were gavage fed 25 mg/kg SNX631-6 (Senex Biotechnology, Inc.) in 30% Propylene Glycol and 70% PEG-400 twice a day. Control mice were gavage fed vehicle (30% Propylene Glycol and 70% PEG-400) twice a day. Treatments began on 5 dpf and continued to 10 dpf or 15 dpf. All mice in this experiment were euthanized at 15 days post fracture. The study design included n = 10 mice per group, however this was reduced to 9 Vehicle, 8 SNX631-6 treated for Cohort 1, 8 Vehicle, 8 SNX631-6 for Cohort 2. Exclusion criteria listed in **Table S4.**

### Euthanasia

Mice were euthanized at endpoints of 4 days post fracture, 7 days post fracture or 15 days post fracture. Euthanasia was performed using carbon dioxide overdose followed by a secondary method of cervical dislocation.

### Tissue Fixation

For micro-computed (mCT) topography and histologic analysis, tibias were immediately removed from the animals and placed into 10% Neutral Buffered Formalin for 48 hours at 4 degrees Celsius. Afterwards, formalin was rinsed out of the tissues using distilled water and the limbs were placed in 70% ethanol for downstream processing.

### Callus harvest and digestion

For single cell RNA sequencing and flow cytometry, tibias were harvested at 4 and 7 days post fracture. Fracture calluses were manually harvested via stereomicroscope dissection from mice at 4 and 7 dpf and digested using a shaking waterbath at 37 degrees Celsius for 30 minutes in 5 mL PBS (+Ca/+Mg) supplemented with 2mg/mL Collagenase P, 2mg/mL Hyaluronidase, and 200 μg/mL DNase. Isolated cells were filtered through a 40 μm cell strainer.

### Micro-computed tomography (mCT)

At 15 dpf, fractured tibias were removed at the knee joint and placed into 10% Neutral buffered formalin for 48 hours prior to transfer to 70% ethanol. Intramedullary pins were removed using small nose needle plyers and fractured tibias were mCT scanned using a Bruker SkyScanner and reconstruction was performed in MicroView. Dragonfly analysis software was used to analyze fracture callus volume, bone volume, tissue mineral content, and bone mineral content. The callus region of interest was determined by manually outlining the callus region every 10 slides throughout the entire callus. The marrow space and cortical bone are selected and removed from the callus area. These images are then interpolated across sections to generate a callus volume. A bone threshold to classify tissue as bony tissue was determined by outlining the calluses, allowing the software to automatically determine the bone threshold for the vehicle samples, taking the average across vehicle samples and re-applying this threshold to all the samples. Bone volume fraction was manually calculated by dividing bone volume by total callus volume. Bone mineral density was determined by dividing bone mineral content by total callus volume, and tissue mineral density was calculated by dividing the tissue mineral content by the bone volume. Analysis was done blinded to treatment or control. Representative callus images were selected from the Dragonfly software.

### Histologic analyses of fracture calluses

For limbs 15 dpf, post mCT scanning, limbs were thoroughly rinsed with distilled water and transferred to Immunocal Decalcifier for 16 hours. X-rays taken using the Kubtec X-ray machine confirmed decalcification. Tissues were paraffin embedded using the tissue processor (Leica ASP 300S) and sectioned in the sagittal plane at 5 μm. Serial sections of the callus were placed on different slides with each slide containing 3 sections approximately 50 μm apart generating an average representation of the callus for each sample. Tissues were deparaffinized using a sequence of xylenes and Ethanol from xylene to 100% ethanol, 95% ethanol, 70% ethanol, to distilled water. Limbs were stained with Weigert Hematoxylin followed by 0.05% aqueous Fast Green, and 0.1% Safranin 0 to label cells bone and cartilage respectively. Fracture calluses were imaged in color brightfield on an inverted microscope (Biotek Lionheart) at 4X stitches. Image files were saved and analyzed in ImageJ to assess the average callus area, cartilage area, and percent cartilage across 3 sections, each 50 μm apart for each sample. A region of interest is established by outlining the callus in ImageJ, the brightness range, saturation range, and hue range and determined to allow for cartilage detection and these thresholds are kept constant across all samples within a cohort. The number of pixels that make up each callus region and the number of pixels that make up the cartilage region within each callus is recorded. The cartilage pixels are divided by the total callus pixels to determine % cartilage area.

For limbs from mice 7 dpf , a mix of males and females were used. The study design included n = 8 mice per group, no mice were excluded (Table S4). Tibias underwent fixation as described above, were placed in 70% ethanol, thoroughly rinsed, and transferred to 20% ETDA for 2 weeks. X-rays taken using the Kubtec X-ray machine confirmed decalcification. Tissues were paraffin embedded using the tissue processor (Leica ASP 300S) and sectioned in the sagittal plane at 5 μm at the center of the callus. Sections were deparaffinized, stained with Weigert Hematoxylin followed by 0.05% aqueous Fast Green, and 0.1% Safranin 0 and analyzed using ImageJ. For all histology analysis, the person performing analysis was blinded to treatment or control.

### Flow cytometry

Flow cytometry was employed to identify and quantify immune cell subsets in fracture callus. Single-cell suspensions of fracture callus cells were stained with live/dead stain (Fixable Viability Dye) and blocked with FcX TruStain PLUS. Cells were then washed, stained in the dark with fluorescently conjugated antibodies for 30 minutes at 4 ° C (**Table S2**), fixed with Fixation/Permeabilization buffer, and passed through a 40 μm filter. A minimum number of 50,000 cells were targeted for each sample. For all flow cytometry experiments, an unstained control, single-stained controls for all targets, and fluorescence-minus-one tubes for all targets were included for robust determination of negative and positive staining in each channel. Experiments were performed in FACS buffer (PBS containing 2% fetal calf serum and 1 mM EDTA). Immune cells were identified by labeling for CD45+ cells. Of the CD45+ cells, macrophages were identified as Ly6G−/F480+/CD11b+; granulocytes were identified as F480−/Ly6G+; dendritic cells were identified as Ly6G−/F480−/CD11c+; T cells were identified as Ly6G−/F480−/CD11c−/CD11b−/CD3+. Samples were analyzed and compensated on the BD LSRFortessa cytometer using FACSDiva (BD Biosciences) software. Data was analyzed using FlowJo v10.9 (BD Biosciences).

### Single cell RNA sequencing (scRNAseq)

scRNAseq (Parse Biosciences pipeline) was used to characterize the transcriptomic differences in early response of Intact and Ischemic fracture calluses at 4 and 7 dpf, representing four conditions. For each condition, we sequenced a single biological replicate made up of 8 pooled calluses (equal male/female). Following enzymatic callus digestion, single cell suspensions were fixed using the Parse fixation kit, frozen down to −80 ° C in media containing 1.6% DMSO, and subsequently submitted to the University of Michigan Advanced Genomics Core for sequencing using the Parse Biosciences pipeline.

Samples were counted using AO/PI stains on the LunaFx7 automated cell counter (Logos Bio). Samples were prepped according to Parse’s instructions for single-cell whole-transcriptome profiling. Cells were thawed and counted on a hemocytometer prior to barcoding. Samples were loaded into Round 1 Plate, by sample, to receive their initial barcode through an *in situ* reverse transcription reaction using barcoded primers. Cells were then pooled from all samples and randomly distributed to Round 2 plate, where they received a second barcode through ligation barcoding using a Ligation Mix provided by Parse. This was repeated to generate a unique barcode for each cell. cDNA was quantified by Qubit and assessed on TapeStation 2200 (Agilent). Final libraries were quantified by Qubit and assessed on LabChip GX Touch (Revvity). Sequencing was then performed in a paired-end fashion (150 bp) on a NovaSeq6000. Processing targeted 12,500 cells/sample with a target depth of 50,000 reads/cell. Raw sequencing data was aligned to the GRCm38 reference mouse transcriptome, and all preprocessing, quality control, and bioinformatic analyses were performed using RStudio (version 4.1.3). Data was imported into Seurat (v4) ^67^ for quality control, filtering, and bioinformatic analysis.

#### Quality control, filtering, clustering, and analysis of scRNAseq data

A rigorous quality control pipeline was employed to remove low quality cells, doublets, and debris (**Fig. S1**). Duplicated genes, empty row names, cells with less than 100 detected genes, and genes expressed in fewer than 10 cells were first removed. Then, low quality cells were removed on the basis of gene/feature expression: 250 < *nFeature* < 4000, 500 < *nCount* < 4000. Cells were kept if they contained log10 genes per unique molecular identifier (UMI) > 0.8 and mitochondrial gene ratios < 7.5%. A global scaling normalization was then applied to normalize the feature expression for each cell by the total expression, and the top 3000 variable features were calculated. Data was then scaled using a linear transformation so that the mean expression of each gene is set to 0 and variance is 1 to account for highly expressed genes, and a regression was employed to remove variance due to cell cycle and mitochondrial gene expression. Seurat (v4) ^67,68^ was used to perform linear dimensionality reduction via principal component analysis, followed by nonlinear dimensionality reduction via Uniform Manifold Approximation and Projection (UMAP) and clustering. Using elbow plots illustrating the proportion of variance in each principal component, dimensions 1–23 were chosen for subsequent nonlinear dimensionality reduction of all cells from all conditions via Uniform Manifold Approximation and Projection (UMAP). Clustering was performed using the Seurat functions *FindNeighbors* (dims 1:23) and *FindClusters* (*resolution* = 0.5), which employ a K-nearest neighbor graph-based approach and the Louvain algorithm^67^.

Cluster gene markers were then derived using the Seurat function *FindAllMarkers* to extract the top 50 markers per cluster. Significant (Padj < 0.05) gene markers were sorted by pct1/pct2 ratio and the top 10 markers were prioritized in identifying each cluster. For each data object, clustering resolution was iterated across a range of the *resolution* parameter (0.2 – 0.7) in the *FindClusters* function to derive a final resolution setting which yielded at least 10 significant gene markers with high pct1/pc2 ratio. Published data, pathway enrichment analysis via Gene Ontology – Biological Processes (GO:BP) (PantherDB^69,70^ and Cluster Identity Predictor (CIPR)^71^ were further used to identify and annotate each cluster based on their top gene markers, as defined above.

#### Subset clustering of immune and stromal cells

Following the identification of broad cell types, we computationally extracted immune cells (clusters 3, 4, and 6) and stromal cells (clusters 1, 5, 8, and 9) from the data object containing all cells from all conditions and all timepoints (Fig. 1B) to identify specific subsets within these populations separately. Clustering parameters were re-established, as described above, to cluster immune cells (dims = 1:19, resolution = 0.7) and stromal cells (dims = 1:19, resolution = 0.5) separately. Cluster gene markers were extracted, and cells were identified by gene marker expression, pathway analysis, and CIPR, as described above. Stromal and immune subsets were named according to prior literature or based on expression of top gene markers. Clusters were identified and labeled according to the expression of top gene markers. Dimensionality reduction and clustering was first performed on all cells from all conditions to identify major cell types, followed by more detailed clustering and annotation of only immune cells (defined as *Ptprc*+) ^72^ and only stromal cells (defined by *Prrx1*^*+*^*/Runx2*^*+*^*/Cdh11*^*+*^*/Postn*^*+*^*/Fn1*^*hi*^*/Col1a2*^*hi*^)^11^ separately.

#### Pathway enrichment analysis

To analyze which biological pathways underpin a set of differentially-expressed genes (DEGs) between two or more conditions, we extracted DEGs using the *FindMarkers* function in Seurat, with *ident1* set as the Intact condition and *ident2* set as the Ischemic condition. A csv data file containing gene names and corresponding log2FC values was then uploaded to PantherDB^69,70^. Statistical overrepresentation analysis was performed utilizing the Mus musculus reference and the Gene Ontology – Biological Processes (GO:BP) annotation. GO:BP results were represented by bubble plots to show the degree of pathway enrichment and the associated Padj/FDR value. Gene set enrichment analysis (GSEA) was used to unbiasedly analyze GO terms in the immune population at 4 and 7 dpf between Intact and Ischemic conditions^73^. GSEA input required gene list, p-value and log2 fold-change which were all taken into account to generate the top GOs. Top GOs were determined according to normalized enrichment scores (NES).

#### Cell-Cell communication analysis

To model cell-cell communication and predict the major cellular signaling pathways from scRNAseq data, we utilized the R package CellChat (version 1.1.3)^24^. A CellChat object was created for each condition and time point separately, and the default CellChatDB.mouse database was used to interrogate ligand-receptor interactions. To assess overexpressed pathways, we utilized the functions *identifyOverExpressedGenes* and *identifyOverExpressedInteractions*. The triMean method was used to infer ligand receptor pairs. A minimum cell number was set to 10 and the probability of the ligand-receptor interaction was set to p < 0.05. The cutoff for contribution score was set to 0.2. Outgoing and incoming signaling pathways were assessed across all Intact cells at days 4 and 7 post fracture. Cell populations were pseudocolored to match the stromal populations in Figure 1D. Riverplots were simplified, and columns added manually based on CellChat output in Figure 3 to show the pathways contributing to each pattern and their contribution score. Full R output of Riverplots are shown in S4 and S5.

Patterns were selected at the peak of the line plot, resulting in 5–6 patterns. The presence/absence of signaling pathways and their corresponding probability values were compared between conditions, utilizing the Intact condition as the control/reference group.

#### Trajectory analysis

Monocle3 (version 1.0.0)^26^ was employed to perform cellular trajectory analysis and unbiasedly assess differentiation-associated genes across genotypes/conditions. We first isolated and pooled all stromal cells at both timepoints for each condition separately (Intact and Ischemic), parameters found in **Table S3**. First, linear and nonlinear dimensionality reduction was performed via PCA and UMAP using the Monocle3 functions *preprocess_cds* and *reduce_dimension* functions, respectively, followed by clustering using the *cluster_cells* function, according to Monocle3 guidelines. Dimensionality reduction and clustering parameters were optimized in a supervised fashion to most closely reflect clustering results from Seurat for each condition. The *learn_graph* function was then used to compute a trajectory within each condition, and the progenitor stromal cells population αSMA+ (*Acta2+*) population was manually selected as the root node in the *order_cells* function, given prior literature demonstrating that αSMA+ progenitors give rise to osteogenic cells in fracture callus ^12^. We then isolated the trajectory segment from the aSMA+ progenitor towards hypertrophic chondrogenic end state using *choose_graph_segments* to subset cells involved in this differentiation pathway. To unbiasedly extract the top genes that exhibit pseudotime-dependent expression changes along this trajectory from aSMA+ progenitor towards hypertrophic chondrocytes, gene expression was analyzed across the subset of cells and those with a q-value < 0.05 and a Moran’s I > 0.25 were selected from the intact trajectory, resulting in 138 genes. The same 138 genes were used in a heatmap for the Ischemic condition. Area under the curve (AUC) as a function of pseudotime was calculated for all 138 genes using the normalized expression in the Intact and Ischemic trajectories. To interrogate the specific undulations in gene expression over pseudotime for the Intact and Ischemic trajectories *plot_genes_in_pseudotime* was used to extract normalized expression and the 95%Cl as a function of pseudotime [using Loess regression]. This was then plotted using GraphPad Prism.

#### Ischemic fibrosis-associated gene expression analysis

We assessed genes differentially expressed in the Ischemic callus at 7 dpf(p < 0.05) and assessed their known role in the literature; we found several determined to be associated with fibrosis and generated a heatmap using their log2 fold change.

### ATDC5 culture and differentiation

ATDC5 cells were purchased from Sigma Aldrich. ATDC5 cells were cultured in growth media consisting of high glucose DMEM with 5% FBS, 1X L-glutamine, 1X antibiotic-antimycotic. To induce chondrogenic differentiation, a standardized protocol was employed^74,75^. ATDC5 cells were grown to 90% confluency, passaged, and plated at 5×10^4^ cells/well in a 12 well plate. After 2 days, the media was replaced with high glucose DMEM (with pyruvate) containing, 1X L-glutamine, 1X antibiotic-antimycotic, 40ug/mL L-proline, 40ug/mL L-ascorbic acid 2-phosphate, 10ng/mL TGFβ1, 1X ITS pre-mix, and 100nM dexamethasone to induce chondrogenesis. Chondrogenic media was replaced every 2 days for 14 days.

To assess the effect of Cdk8 inhibition on chondrogenic differentiation, cells were treated with either vehicle (PBS) or fresh 400 nM Senexin B (resuspended in PBS) with each media replacement. ATDC5 treated with SNX631-6 at 0 nM, 25 nM, 50 nM, 100 nM, 200 nM, and 400 nM in the presence of TGFb1 were similarly treated with new media containing SNX631-6 every 2 days for 10 days. At harvest, cells were lysed with TRIzol, RNA was extracted, and chondrogenic gene expression was assessed via quantitative real-time PCR (qPCR). All primer sequences are listed in **Table S5**. All ATDC5 experiments were repeated 3 independent times with 3–4 technical replicates each time.

### Primary human mesenchymal stromal cell (hMSC) culture and differentiation

Donor hMSC were provided from Case Western University for chondrogenic pellet assays and osteogenic assays through a NIH/NIBIB funded P41:1P41EB021911. hMSC were provided from Texas A & M Health Science Center College of Medicine Institute for Regenerative Medicine at Scott and White through a grant for ORIP of the NIH #P400D011050 for gene expression analysis in hypoxic conditions.

For chondrogenic differentiation, hMSC were recovered from liquid nitrogen into low glucose DMEM (Gibco, cat. 11885084) containing 10% FBS, 1X antibiotic-antimycotic, 10ng/mL fibroblast growth factor 2 (FGF-2). Cells were grown to 90% confluency prior to passage and expansion. hMSC underwent trypsinization and were centrifuged at 500g for 5 minutes. Post centrifugation, cells were resuspended in chondrogenic media containing DMEM, 1X antimycotic, 1X non-essential amino acids, 40ug/mL ascorbic acid 2-phosphate, 10ng/mL TGFb1, 1X ITS pre-mix, and 100nM dexamethasone. To generate three-dimensional pellets, hMSC were plated at 2.5×10^5^ cells/well in 96 v-bottom plates, as described^75^. Plates were spun down at 500g for 5 minutes to condense the cells and allowed to rest for 3 days to form pellets. For the duration of the experiment, pellets were treated with either Vehicle (PBS) or 400 nM Senexin B at each media exchange every second day up to 14 days. Pellets were then harvested for either gene expression analysis, histological analysis, or quantification of sGAGs via the DMMB assay (details below). All experiments were performed in three unique hMSC donor lines, and each assay was conducted in 2–3 technical replicates.

For osteogenesis, hMSCs were cultured in a monolayer in osteogenic media (αMEM, 1% antibiotic antimycotic, 1% l-glutamine, 1X insulin transferrin selenium, 25 μg/mL l-ascorbic acid 2-phosphate, 5 mM beta glycerophosphate) (**Table S2**) for 10 days. Cells were exposed to BMP-2 (200ng/mL) for the first 4 days and treated with either vehicle (PBS) or 400 nM Senexin B at each media exchange. Cells were fixed and stained with Alizarin red to assess mineralization.

For hypoxic study, hMSC were cultured in a monolayer in serum free media (αMEM, 1% antibiotic antimycotic, 1% l-glutamine, 1X insulin transferrin selenium, 25 μg/mL l-ascorbic acid 2-phosphate, 5 mM beta glycerophosphate) (**Table S2**) in a H35 hypoxystation (Don Whitley Scientific) at 1% oxygen tension or under normoxic conditions (20% oxygen) for 72 hours. Cells from 5 unique donors were harvested for RNA and analyzed via qPCR for gene expression of *CDK8* relative to b2-microglobulin (b2M) housekeeping gene. Primer sequences are listed in **Table S5**.

### Gene expression analysis via qPCR

For ATDC5 cells and monolayer hMSC, TRIzol was added to monolayer cultures to lyse cells. hMSC pellets were flash frozen, stored at −80C until analysis, and then homogenized in TRIzol using a manual tissue grinder. Each hMSC pellet condition had 2–3 technical replicates comprised of 3 pellets pooled together to increase RNA quantity. All samples then underwent phenol-chloroform RNA isolation. Spectrophotometry was performed using a Nanodrop (ThermoFisher) to calculate RNA concentration and assess purity. A standard 500 mg was taken from each sample to perform reverse transcription using a High-Capacity cDNA Reverse Transcription Kit (Applied Biosystems). A QuantStudio5 Real-Time PCR System (Applied Biosystems) was used to perform qPCR. The housekeeping genes, GAPDH or b2M, was utilized to normalize the genes of interest. Control samples were set to 1 using the 2^−ΔΔCt^ and all samples were normalized to control. All primer sequences are listed in **Table S5.**

### Glycosaminoglycan and DNA quantification

Chondrogenic pellets were harvest by flash freezing in liquid nitrogen and stored at −80C until analysis. After thawing, samples were immersed in buffer containing papain (0.025mg/mL), L-cysteine hydrochloride (0.315 mg/mL), EDTA (0.744 mg/mL), and sodium phosphate (0.0071 mg/mL). Samples were then heated at 60 degrees for 16 hours. Samples were vortexed and homogenization was visually confirmed. Di-methyl methylene blue dye (containing 1,9 Dimethylene blue, glycine, NaCl) (pH 1.5) was added to each sample at a ratio of 1:20 and absorbance was immediately quantified at 525 nm and 59 5nm using a spectrophotometer (Synergy HTX, Biotek) as previously described (61). Data was calibrated to a chondroitin sulfate standard curve to quantity sulfated glycosaminoglycans. Samples were then normalized to their respective vehicle control for each donor to assess total changes in glycosaminoglycans. The Quant-iT PicoGreen dsDNA assay was utilized to further quantify DNA content in homogenates, utilizing dsDNA standards as a calibration. sGAG data was then normalized by DNA content to control for cell density.

### Histological evaluation of chondrogenic pellets

Chondrogenic hMSC pellets were collected into 10% neutral buffered formalin for 16 hours, transferred to 70% ethanol, and paraffin processed and embedded. Five micron-thick sections were cut and stained with Alcian Blue (1% in 3% Acetic Acid). In brief, slides were deparaffinized and rehydrated in a series of xylene and ethanol. Slides were rinsed in MilliQ water, placed in 3% acetic acid, and incubated in Alcian Blue staining solution. Slides were then rinsed in MilliQ water and incubated in Nuclear Fast Red Solution. After staining, slides were rinsed in MilliQ water, dehydrated through a series of ethanol to xylene and coverslipped using Cytoseal. Stained sections were imaged by brightfield microscopy (Lionheart, Biotek) at 10X magnification and stitched together to capture a full pellet.

### QUANTIFICATION AND STATISTICAL ANALYSIS

All group data are presented as mean ± 95% confidence interval and P < 0.05 or P_adj_ < 0.05 was considered statistically significant throughout comparisons. For continuous data, normality and equal variance were confirmed via Shapiro-Wilk and Levene’s test, respectively and compared using either independent or paired two-tailed t-tests or ANOVA, as applicable. In cases where normal distribution was not achieved, the non-parametric Mann-Whitney test was used. In cases of nonequivalent variance, a Welch’s corrected t test was applied. A sample size of 8–10 mice was chosen for uCT and histology based on power analysis of previous published studies performed by our lab. All bioinformatic analyses utilized default statistical approaches integrated into the R packages Seurat, Monocle, and CellChat as well as statistical enrichment or statistical overrepresentation testing in PantherDB. All bioinformatic analyses employed statistical adjustments for false discovery rate across factors such as cell and gene numbers, and P_adj_ was used throughout. GraphPad Prism and RStudio were used to perform statistical analyses and generate figures.

## Data Availability

All data and materials information are available upon request. Sequencing data will be made available through the NCBI Gene Expression Omnibus (GEO) prior to manuscript publication (GSE292578). All code is available upon request.
